# Using participatory video to generate active agents of change at community level to address the drivers of antimicrobial resistance in two settings in Nepal

**DOI:** 10.1186/s12889-024-21181-6

**Published:** 2025-03-25

**Authors:** Nichola Jones, Abriti Arjyal, Rebecca King, Jessica Mitchell, Ines Soria-Donlan, Sushil Baral, Paul Cooke

**Affiliations:** 1 University of Leeds, Leeds, UK; 2 Herd International, Kathmandu, Nepal

**Keywords:** Community engagement, Advocacy, Filmmaking, Antimicrobial resistance, Participatory video, Co-production

## Abstract

**Background:**

We present a community-based videomaking project that used Participatory Video (PV) to co-produce community-led resources to address the issue of antimicrobial resistance in Nepal. Specifically, this paper highlights the potential of PV as a way of generating community champions that can become active ‘agents of change’.

**Methods:**

A total of 20 participants took part in PV workshops in Nepal across two settings; one urban and one peri-urban site. Participants were trained in video production and took part in interactive learning sessions on AMR. Participants were supported to create and showcase their own videos on AMR in their community. All workshops were recorded and a series of focus group discussions and interviews were undertaken to evaluate the project.

**Results:**

Participants considered PV to be a positive experience, both in terms of personal development and their ability to understand and address community-level drivers of AMR. They emphasised how the project helped them to become proactive in addressing AMR and also to be ‘seen’ by policy makers and other members of their community who they did not feel would generally take notice of them. Conversely, policymakers, as well as other members of the participants’ communities, were impressed by the quality of the work produced, which, in turn, made them pay attention to the messages communicated in the videos.

**Conclusions:**

CARAN highlights the potential of PV as a way of creating community-level champions to help address the drivers of AMR. More work is required to understand the longer-term value of creating such champions.

**Supplementary Information:**

The online version contains supplementary material available at 10.1186/s12889-024-21181-6.

## Introduction

In this paper we present the case study of ‘Community Arts Against Antibiotic Resistance Nepal’ (CARAN), a video production project that used short videos as a way of creating bespoke community-appropriate public health tools to address antimicrobial resistance (AMR) and to communicate community perspectives in this issue to policy makers. We outline the potential of such engagement to create effective, targeted communication tools that can ‘cut through’ with intended audiences. Specifically, we explore the utility of so-called ‘Participatory Video’ (PV), focussing on the ways in which PV can generate local-level advocates for behaviour change with regard to AMR. In an earlier paper we presented an overview of the CARAN project, looking at the how we delivered our initial workshops and the types of videos that were produced [1]. In the present paper we give a summary of this aspect of the project. However, our focus in this paper is on how the use of PV supported active community engagement through which participants were able to see themselves as agents of change, rather than as passive participants in a public health education project [2]. Moreover, we show how this, in turn, not only changed the way that participants saw themselves, but also how others saw them, and how this might have helped to magnify the wider impact of PV with relevant stakeholders. Central to this was the project’s dissemination strategy. This is an aspect of PV that is often neglected in the literature, which tends to focus on the initial production phase of a project and so does not tend to explore the longer-term potential of such work to effect change and the role that participants themselves can play in this process [3].

PV is an arts-based methodology that utilises a form of ‘co-production’ to generate videos with community groups, aiming to actively engage participants in the exploration of topics that are important to them [4]. The origin of PV is frequently traced back to the 1960s and the Canadian Film Board’s ‘Challenge for Change’ programme (Baker et al., 2010). Interest in the method has grown exponentially since then, initially with the development of lightweight video equipment in the late 1960s and 1970s, and even more so with the advent of social media in the 2000s and the growth of web platforms such as YouTube, Instagram and TikTok [5]. The aim of PV is generally twofold. On the one hand it is designed as a tool for community-level self-reflection, allowing participants, through the process of making videos, to reflect on a particular issue they face as a community. On the other, by screening the videos produced the community can generate further discussion with relevant stakeholders in order to help inform community-appropriate solutions to this issue. The literature on PV repeatedly emphasises its potential to effect positive social change, particularly on participants themselves [6]. It is frequently endorsed for the ways in which it can — if designed carefully and in a manner that is cognisant of specific cultural dynamics at play in a given context — diminish traditional hierarchies between researchers and participants and can create spaces of learning [7], playing a particularly important role in supporting and amplifying the voices of marginalised communities [8]. There is generally less detailed discussion of the broader impact of such work and how participants, and the videos they produce, can effect wider social and cultural change [3]. This is, of course, difficult to quantify. In this paper we look at the way people engaged in CARAN saw at least the potential for the project to develop this kind of broader impact.

Antimicrobial resistance (AMR) is a major threat to global health, food sustainability and security and socio-economic development. Recent findings suggest that AMR was directly responsible for over 1.2 million deaths in 2019 alone [9]. Furthermore, deaths related to AMR in 2019 exceeded 4 million, making it the second leading cause of death globally, highlighting our growing inability to prevent and treat multiple common infections [9]. AMR is a critical concern in Nepal and is primarily caused by the overuse, misuse, and, indeed, underuse of antibiotics [10]. Although much progress has been made to reduce morbidity and mortality, infectious diseases that are reliant on antimicrobials for their treatment, such as tuberculosis, respiratory infections and diarrheal diseases, remain top causes of death in the country [11]. The global response to AMR has resulted in multi-sectoral initiatives and global guidelines that stipulate the critical actions required [12–15]. Both prescribers and users, it is argued, have become too dependent on antimicrobial agents to manage disease, and there is widespread lack of understanding of the dangers of antimicrobial resistance and its impact. The need for public awareness and/or public education on AMR is highlighted in most international guidance. Moreover, it is increasingly acknowledged that to tackle AMR it is critically important to go beyond simply raising awareness through public health campaigns [16]. Direct, active engagement with the communities most impacted by AMR is required. Community generated solutions to local issues around antimicrobial misuse are essential to effect behaviour change [17–20]. However, active community engagement to address AMR remains an under-used and under-researched strategy for addressing AMR globally and receives little mention in global policy. As Matthew et al. put it, ‘Global and national AMR mitigation efforts have been largely top-down with sub-optimal impact downstream, necessitating a complementary bottom-up approach where community engagement is prioritized’ [20].

In this paper we present the ways in which we used PV in CARAN to generate this kind of ‘bottom-up’ active community engagement, the first project to adopt this approach in AMR research globally. In particular, we explore the multiple ways in which the use of PV can shift how participants understand and value their knowledge about the community-level drivers of AMR, as well as the extent to which this work can change perceptions of how other stakeholders value this same knowledge. Crucially, however, we also point to the potential limitations of such approaches and of community-produced videos, calling for a carefully curated, ‘situated’ form of engagement that remains rooted within the community of its production. This is key if we are to ensure that this material is shared appropriately and generates useful community-level debate about AMR, rather than potentially spreading misinformation or simply being ignored in the white noise of the internet and social media.

## Methods

**Fig. 1:**
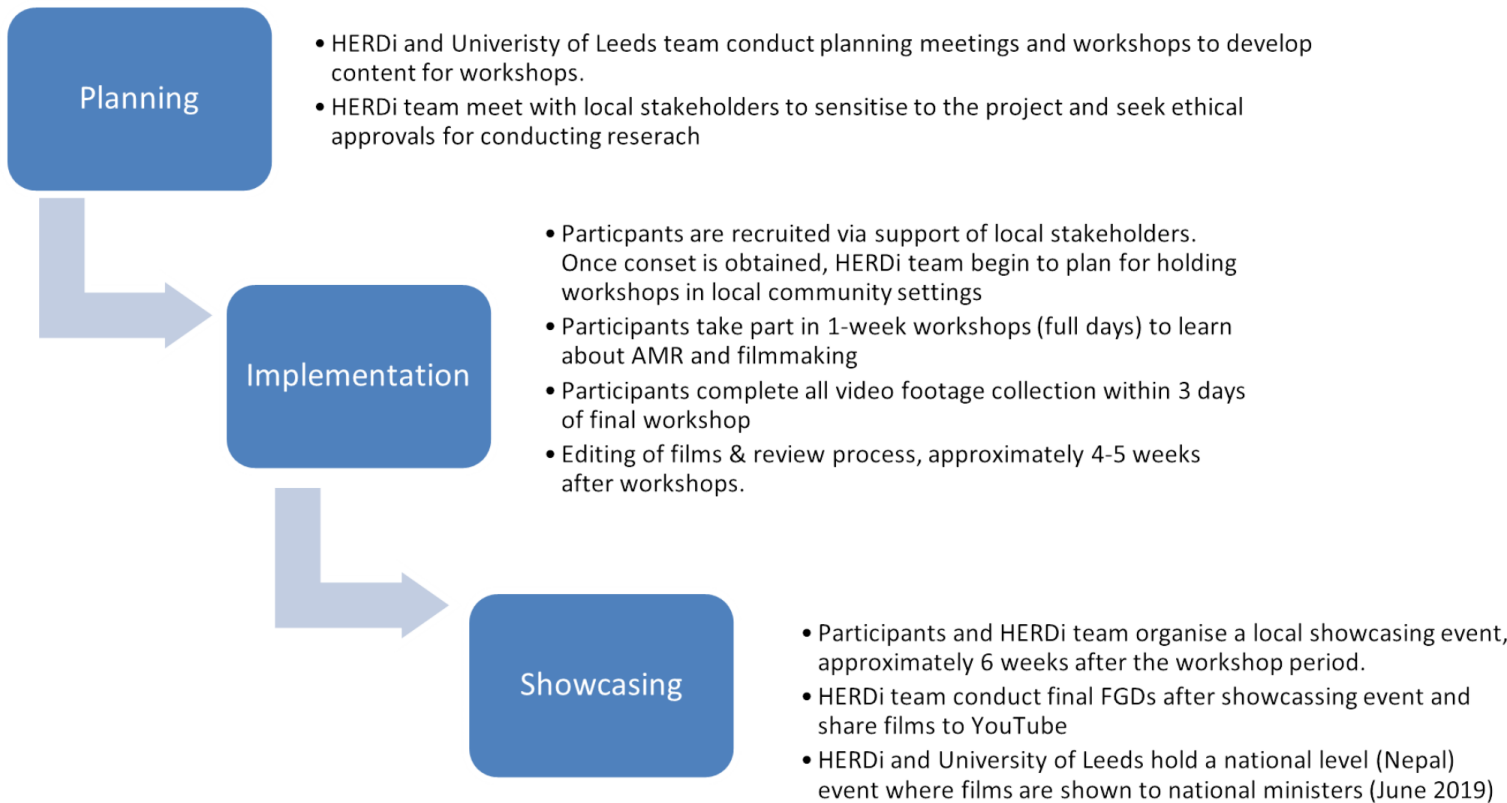
Overview of the CARAN project

The CARAN project was a collaboration between public health professionals, policymakers, creative arts practitioners and community participants. The planning and implementation of this project were conducted over an approximately one-year period in 2017–2018, with fieldwork activities conducted over a concentrated two-month period of that time. The research team had a wide range of research backgrounds in Medicine, Public Health, Anthropology and the Humanities, drawn from the University of Leeds in the UK and HERD International (HERDi), a Nepal-based public health research agency. Figure 1: Overview of the CARAN Project below offers a diagram of the overall project process:

### Description

Figure 1 shows a flow chart diagram of the overall CARAN project process; planning, implementation and showcasing stages are illustrated. HERDi (HERD International) is a Nepal-based research and development organisation working in public health across Nepal. University of Leeds team members are based across the School of Medicine and School of Arts & Humanities. The term AMR refers to Antimicrobial Resistance. The term FGD refers to Focus Group Discussions.

### Setup

Initial co-ordination meetings were held with stakeholders from each municipality to sensitise them to the study and seek permission to conduct the study. Once municipalities were confirmed, specific study sites were chosen; one peri-urban site in the Chandragiri municipality and one in an urban settlement in Bhaktapur, Lokanthali. Within study sites, local stakeholders (e.g. local health workers) acted as gatekeepers to the participants. These gatekeepers shared information about the project locally for recruitment and reached out to specific community members where appropriate. gatekeepers shared information sheets and contact details of the HERDi team for the formal recruitment processes.

### Sampling strategy for project delivery

A total of 20 participants—10 participants from each site—were selected to take part in the project. The participants represented different social demographics such as age, occupation, education level and gender within each study site. No previous experience of video production or existing knowledge of AMR was required, simply an interest in learning more about both. An additional file provides a table with further participant characteristics (see Additional File 1).

### Participatory video production process

Participants attended a series of five workshops where facilitators and participants explored issues relating to AMR, as well as learning the basic principles of filmmaking. Facilitators were members of the HERDi team, speaking in Nepali. By the end of the fifth day, participants had shot all the footage for their video. Final editing of the videos was conducted by a videographer in the HERDi team, with participants reviewing rough cuts of the footage before giving final approvals. The editing process took approximately six weeks.

### Showcasing

The production process was followed by two local showcasing events, designed to share the videos made by participants. During this stage, recruitment of audience members was directed by workshop participants. Workshop participants were encouraged to invite friends, family and community members and share the showcasing information widely to invite as many local people as possible to each event. Participants also worked with facilitators from the HERDi team to invite appropriate policymakers, as well as other relevant stakeholders from beyond their immediate community that they might not have direct access to. Attendees included municipality mayors and deputy mayors, ward chairs, local health professionals as well as providers of wider local amenities (including youth leaders, local teachers, health professionals). Over 200 people were invited to each event with well over 100 attending each.

### Evaluation of the PV process

For the purposes of evaluating the project, all the workshops were recorded (video files later transcribed to written documents for analysis). This was complemented by reflective notes from facilitators. Two focus groups were conducted with people who attended the showcasing events. In Chandragiri 12 people took part (4 female and 8 male). In Lokanthali there were two (one male one female). Participants were asked about what they took to be the key messages from the videos, what they thought worked well during the production process, what they thought could be improved and how they would like to see the project taken forward.

### Participant sampling strategy for post-project evaluation

In addition to material produced during the project workshops, two follow-up focus groups were conducted a month later, to which all project participants were invited. Six participants attended the first focus group, held in Chandragiri (five female and two male) and nine the second, in Lokanthali (four female and five male). These discussions looked at all aspects of the project, from their initial participation through the video production process to their involvement in the final showcasing events. For each stage in the process the participants discussed what went well, what was challenging and could have been better if we were to repeat the project. See Table 1 below for information on the types and sources of data.

**Table 1:** Evaluation data summary

Type of data	Source	When collected	Total
Reflective notes	HERDi facilitators	After each workshop	10
Workshop activity transcripts	Workshop participants	During workshops	6
Focus group transcripts	Workshop participants	One month after showcasing	2
	Showcasing audience members	At the showcasing events	2
Interviews	Policy makers	In the weeks following the follow up focus group discussions	3

The focus groups were followed by two interviews with two local and one national policy maker who attended the Chandragiri showcasing event. Policy makers from Lokanthali were contacted but no times for interviews could be found. In a wide-ranging, unstructured ‘discovery’ style interview, the two policy makers were asked about their impressions of the showcasing events, the videos produced and what (if anything) they thought could be done with the videos in the future.

Focus groups and interviews were audio recorded, and later transcribed into word documents for analysis. Field notes were taken by facilitators on observations from workshop activities, reflections on the implementation of activities and points for development in subsequent workshops. All data was collected at the study sites in Nepali and translated from Nepali to English to facilitate the inclusion of the UK team in the data analysis stages.

#### Ethical issues pertaining to human participants

The topic and methods of the CARAN project were not considered to be sensitive. However, researchers were very aware of the importance of facilitating the process adequately to ensure that the videos produced by participants were factually accurate in terms of AMR messaging. Researchers were careful to ensure that workshop participants understood the key messages of AMR (transmission, scale, drivers etc.), while also ensuring the films were reflective of the specific barriers to the appropriate use of AMR that participants wished to highlight.

#### Method of data analysis

This paper focusses on the PV process, the impact of the project on participants in terms of skills development and empowerment, and how this, in turn, highlights the potential of PV as a form of advocacy that can amplify marginalised voices in larger AMR conversations. The importance of this dimension of the project emerged during our initial examination of the data outlined above.

### Data analysis methods

Thematic analysis was applied to the CARAN data to capture the experiences and attitudes of workshop participants and wider community members and policymakers who attended project showcasing events. After an initial exploration of the data, it became clear that there were points of correspondence between CARAN and other PV projects that members of the team had been involved in. Thus, the team decided to examine the data with an eye on its relationship to the wider literature on PV (Cooke et al., 2020; Cooke et al., 2022; Sarria-Sanz et al., 2023). From this literature, we expected to see themes emerge around participant experiences and perceptions of their own knowledge, the process of co-producing messages with community members and the use of the videos produced as advocacy tools. These themes were used to categorise findings. Further themes and sub-themes came from iterative thematic analysis on close readings of the data. Following this process, three independent researchers divided the transcripts among themselves and coded the data using NVivo Software, a qualitative data management and analysis software package. Coding again progressed iteratively, with continuous discussions and reflections across the research team to maintain consistency and the development of new codes, as needed.

## Results

### Participant experiences

Workshop participants reflected on their experiences throughout the data collected. This they considered to be overwhelmingly positive, both in terms of their own personal development and their knowledge and ability to understand and address community-level drivers of AMR. Within this overarching theme, three subthemes emerged. Participants repeatedly emphasised (i) *their sense of pride* in their participation (ii) how the technical skills they learned in participatory filmmaking helped them to grow in *confidence*, in turn (iii) *empowering* them to make a significant contribution to their community.

Describing the participation of the local mayor in the community showcasing event in Chandragiri, for example, one participant spoke for many when he talked of how ‘proud’ he was of his participation:

*I feel that the things that the mayor said and the personal experiences that he shared, listening and understanding those things, I have found that we have worked a lot… at that time I could feel the worth of the effort that we had put in. I felt that we had done a good job… I also realized that our effort was seen and appreciated. P7, Chandragiri FGD*.

Here, the participant links feelings of pride with the recognition received from the local mayor, with being ‘seen’. This is a connection made frequently in the data. Participants’ experience of pride was often related to a sense of recognition and increased respect from their community, a sense of respect that helped to grow their confidence to see themselves as active members and contributors in their communities.

Although motivated and excited to participate, participants were also frequently nervous at the outset of the project and were not sure if they would be up to the tasks involved. However, this invariably changed as the training programme and interactions with facilitators progressed:

*It was hard as long as we did not [know] how to do it. We became confident as we kept working on it. Looking at the video, I felt that it would be easier once we become confident. I was happy looking at the video with the message that we should not be careless about the use of antibiotics as I could see that I was growing confident towards the end. P7, Chandragiri FGD*.

*It was a bit difficult taking all the equipment out in front of new people. They would be wary of the camera and the sound equipment… We had to pay attention to all the details that we were taught such as attaching the jacks and how to conduct an interview. At the end of it, I felt that I had done something big. P3, Chandragiri FGD*.

Participants no-longer felt ‘small’ (*P3, Chandragiri FGD*). They were able, as this participant makes clear, to make a ‘big’ contribution that ultimately went beyond the specific filmmaking skills they were learning. Hosting one of the showcasing events, for example, put one participant in a position of authority ‘I had never [had] before’ (*P1, Lokanthali FGD*), giving them the confidence to express opinions about important (‘big’) public health topics such as AMR (*P3, Chandragiri FGD*).

### Process of co-production

The growing sense of confidence felt by participants was strongly related to two subthemes: the *participatory* nature of the project and the ways in which *facilitators supported* participants to take ownership of the work, so that they genuinely felt that it was a collaborative process of co-production:

*In previous training I attended (outside of CARAN project), they had me writing down the things we discussed on the board with a marker. We were taught things like they do in the school. We wrote it down in our copies and that is where we were limited to. But the difference here was that we learnt how to practically implement it. P3, Chandragiri FGD*.

Of pivotal importance in this process was the role of the facilitators. This began, participants suggested, with the selection process, in which participants clearly felt a sense of empowerment by the trust the facilitators had put in them through their selection, as well as responsibility for sharing the knowledge they were gaining with their wider community:

*We were chosen for this program assuming that we could teach the people in the community about it. The seniors and the concerned people did so according to the information that they had. P3, Chandragiri FGD*.

*I think that they expected us to be able to teach the rest of the people in our community. I think that is the reason why they gave us a call to come here. P2, Chandragiri FGD*.

The facilitators were repeatedly cited as helping the participants to engage with their wider community, helping them to see themselves as ambassadors for the project and its aims:

*It was very difficult for us to approach and interact with new people. But we were able to perform those activities through your [indicating facilitators] assistance. We would never have been able to accomplish what we have done without your [indicating facilitators] help and guidance. P3, Chandragiri FGD*.

*It was better that you [indicating facilitators] were facilitating us. We used to feel that we might have made mistakes but after your visits and feedbacks, we used to feel that the film will be good. P7, Lokanthali FGD*.

Interestingly, the facilitators themselves at times felt that they might have been too interventionist in this regard. However, the participants were keen to disabuse them of this:

*Facilitator: We used to continuously make phone calls as well as make personal visits to you during the filming period. We used to provide feedback and make suggestions to you. Did you feel offended by this or did you find it useful? Or did it make you feel overburdened and want to quit?*

*P7: No, we didn’t feel anything as such. Rather it made us even more determined to work harder to accomplish the task with the facilitation that you [referring to facilitators] offered.*

*P9: At times we used to receive phone calls from you [referring to facilitators] which helped us to become even more determined to effectively complete the task as we used to feel that not only us but you were also making equal effort in that. Lokanthali FGD*.

### Use of videos for advocacy

Showcase-event audience members were overwhelmingly positive about the videos. They praised how ‘*clear’* (*Participant in Lokanthali showcasing FGD) and ‘informative’ (Participant in Chandragiri showcasing FGD)* the messages contained in the videos were and praised how the videos strongly provoked the audience to use antimicrobials rationally:

*They now know that they are not supposed to simply buy the medicines that they want and consume it carelessly. People have learnt that going to the medical and getting some cetamol for their headache is not a proper practice. Participant in Lokanthali showcasing FGD*.

The videos seemed particularly to resonate with the audience because they were made by people like them and not professional actors:

*We also learnt that it is not just the professional actors that can act in the films. I experienced that the people in the community can also do anything that we wanted to. Participant in Chandragiri showcasing FGD*.

And the scenarios generated seemed directly applicable to their own lives:

*I felt that it was an incident taking place somewhere in real life. The condition is still the same in the villages. Participant in Chandragiri showcasing FGD*.

It was clear from feedback, as can be seen above, that the videos raised awareness of the key generic messages present in the global guidance on mitigating AMR. Showcasing audience member repeatedly mentioned, for example the point made above, the need to only take medicine as prescribed by medical professionals. However, it was also interesting to see how members of the audience also nuanced these messages with reference to local issues, suggesting, for example, that the films had shown them that ‘We should not blindly trust the *jhankri* [traditional faith healers] (*Participant in Chandragiri showcasing FGD*) (for further discussion of this aspect of the films see Cooke et al., 2020). Finally, there was also a strong emphasis in the discussion on the need for the videos to be shared more widely. ‘It would be better if it was showcased in every village’ (*Participant in Chandragiri showcasing FGD)*, was a comment that was repeated in various forms throughout the showcasing FGDs.

A key element within the advocacy theme was the sense of ownership over the films. Showcasing audience members saw their own experiences reflected in these videos made by members of their community. For the video-makers themselves, they similarly saw their work as a way of directly capturing the reality of their lived experience. However, they also saw the films as a way of going beyond their immediate communities, amplifying the comments by audience members that the videos should be more widely disseminated. Within the theme of advocacy, three sub-themes emerged from the focus group discussions with participants: knowledge sharing within the community, knowledge sharing beyond the community and the role of community members as experts.

Turning firstly to the ways in which participants valued their ability to share knowledge within their communities, participants often referred to how the videos allowed them to highlight what they had learned about AMR with members of their community, in particular family members. Here participants highlighted the way they specifically sought to *curate* the video. Participants described sharing excerpts from their work, alongside having discussions with friends and family, during which they brought in further knowledge as a way of raising awareness about AMR. Notable is the sense of responsibility in doing this expressed by participants, seeing the project as a way of supporting AMR education both through their videos and their actions:

*P6: My uncles were unable to [attend the showcase event] because of their busy schedule. I had a video sample with me which I showed my uncles. They told me that I had done well. The thing is that all of them will not know everything about the antibiotics. So I shared [the video] along with the additional information that I had with my family. They said that they learned some new things. I told my uncles, they might tell their friends about it. I think that that is how the information will flow.*

*P3: I felt that the responsibilities that we have in the community should start from us.*

### Facilitator: what about the rest of you?

*P1: I liked it. I felt that if we would take the lead and handle it then the rest would also follow us. We have to be an example to others.*

*P9: When we start working and taking the lead, the rest of the people will also consider us to be an example and follow us. I think that people will follow what we do. Lokanthali FGD*.

Some participants emphasised their role within the community to generate awareness about rational antimicrobial (and in particular in this context antibiotic) use and the value of the videos in supporting this endeavour. Videos, participants suggested, had the potential to create a lasting impression on their community:

*Most people are unaware about what antibiotics actually are. They are very careless. We have gotten a chance to look at our life in detail because of the 5-day training that we have received from your organization….There are 9 participants here, if we were to divide three people into each group and make people aware about these things in our community itself, then others will also pay more attention to us rather than just a person talking about it. When we share the things that we have learnt from this organization, we will teach around 100 people about it. Amongst them, at least 60 people will make changes in their behaviour. P3, Chandragiri FGD*.

As can be seen from the comments by audience members, an important moment in the way the participants conceptualised the project’s awareness-raising potential was the showcasing events that they organised. These events took considerable effort to put together and were central to the sense of empowerment the project generated in participants, who could see that they had the ability to mobilise people in ways they had not previously considered:

*P9: I thought that the people mightn’t attend the program as expected; however, many people attended the program so, I felt very happy. […]*

*P4: I also felt the same thing. I felt that the result that we obtained was more than what we have imagined.*

*P7: People were watching the film with full concentration. So, I thought the people might have liked our program so their focus didn’t drift away from the film throughout the showcasing. Chandragiri FGD*.

*P7: Yes, the DIAL’s [organization that works for slum children] ma’am mentioned that she had never thought that we were being part of this big cause. She acknowledged that we had done a good work.*

*P3: [says excitedly] She mentioned that we had accomplished such a big thing and appreciated our effort as well. Lokanthali FGD*.

In the FGDs, workshop participants reflected on the reception of the videos by their communities. Some reported that audience members had requested access to the videos again. Participants described that their families had also wanted to re-watch videos and had asked if they could access them online. However, crucially, the showcasing events also highlighted to participants how the videos could be used to engage people beyond their immediate communities in conversations about AMR:

*P3: There was an uncle who had suggested that we bring [the videos] to the mainstream media to raise awareness. […] People might get bored when they are informed about it in person, but they would think about it ten times if they could see it visually. Chandragiri FGD*.

The wider educational value of the videos was also highlighted by some of the policy stakeholders who came to the showcasing events. In an interview, the Mayor of Chandragiri, for example (and echoing the comments of other audience members), talked of the videos as ‘a beautiful document’ that he would like to ‘present in all the wards of our municipality. This, I believe, will result in the increased rational use of antibiotics in general,’ the Deputy Mayor subsequently committing to lobbying for resources to achieve this. Indeed, one national policy maker from a national political party went further still, suggesting that this should be a national programme *(Interviews with policy stakeholders).*

The enthusiasm for the videos amongst policy makers was clearly highly motivating for participants and helped them to see a broader value in their work beyond their particular communities:

*P1: Similar to what deputy mayor mentioned, I think showcasing this documentary among the people of at least 3 wards at a time would be an effective step. Even though we are unable to make this kind of documentary in other places, showcasing of this very documentary among other wards would be very effective. Chandragiri FGD*.

For the policy makers, however (as already noted with regard to the wider showcasing audience), it was also clear that the broader power of these videos was rooted in their authentic presentation of local knowledge, on the one hand, and the ability of local people to articulate this knowledge on the other:

*The plans and policies are supposed to be made at the ward or the municipality. That is why the local level representatives that are there at the ward level should also know about these things. So all the elected representatives and advisers of different committees within that ward or the municipality should also be shown [these videos] at least once. They will realize that it is important and that they need to make plans and policies that address it.*

### Interviews with policy stakeholders

The power of the project for this policy stakeholder at this showcasing event was the way the videos highlighted the local expertise of participants. They have knowledge that the policy community does not have and, by implication, can only get access to through this kind of engagement. The creation of videos, which can be shown in different contexts, and ultimately curated in different ways for different audiences, provides an excellent opportunity for such engagement.

## Discussion

Within participatory research broadly, and PV research specifically, much consideration has been given to engagement as a means of evidencing empowerment. One key limitation here is a potentially idealised view of PV as a means to fully empower participants and marginalised communities through an often western lens of liberation and emancipation [21]. In this discussion, we share what we consider to be evidence of engagement but are cautious not to consider this as an immediate precursor to ‘empowerment’ within this community. We do however suggest that, in creating tools for communication and in encouraging participants to speak directly with different stakeholders, PV has the potential to begin the process of empowerment in this setting.

Reflections from workshop participants suggest high levels of engagement with both the process of PV and the continuation of awareness-raising locally after the end of the initial video-production and showcasing phases of the project. Although difficult to measure [22], ideas of engagement are displayed by participants and wider community audiences. Within community settings, participants described showing films and discussing what they had learned in workshops, highlighting the ways in which they sought to curate the videos to maximise their educational potential.

While being rooted in their lived experience, the filmmaking process helped participants to understand the broader value of this experience in triggering wider discussions on ‘big’ issues such as AMR. Through the process of curation alluded to by participants, it is clear they do not feel that the videos alone are enough to provide a coherent way of advocating for better AMR awareness locally. Nonetheless, they clearly consider them to be valuable resources. Participants reflected, during workshops, on the potential of using the videos and information learned during the project, if carefully contextualised, beyond the intervention. The production process itself was also considered to be valuable in the way it helps participants to develop into active champions for this topic, allowing participants to see that their knowledge can make a genuine contribution to local, or indeed national efforts to address AMR.

CARAN suggests that Participatory Video methods have the potential to increase participant confidence in discussing complex health issues [6, 23, 24]. Participants in this study expressed heightened levels of confidence after having seen themselves on screen, a process of self-reflexive empowerment that enabled those participants to see themselves as capable of advocating for change in their community. The community, on watching the films, reflected that seeing local people on the screen not only provided education on AMR, but created a new connection to this issue they had not previously had. Feedback from audience members suggested that they were able to see the issue of AMR as locally applicable and important, as well as being able to accept the messages being delivered more readily because they were being generated by local people. Moreover, audience members, both from the community and policy makers, were impressed by the achievements of the participants, not necessarily expecting them to be able to make videos that could communicate complex messages so effectively.

Participatory video aims to reduce hierarchies in research and empower participants. Terms like ‘empowerment’ in health research are inherently difficult to define [25–27]. Indeed, they often (unintentionally) draw on patriarchal or colonial power dynamics between high income or ‘developed’ countries and low-income or ‘developing’ countries [27–29]. Incorporating PV in this setting aimed to challenge traditional power dynamics in the research process, an essential component in generating ‘empowerment’ for participants [28]. The project sought to highlight how participants could bring their expert knowledge on the local context to bear on the issue of AMR in a similar manner to the way the researchers might bring their ‘scientific’ knowledge on the topic or their technical knowledge of video production. This process of co-creating video outputs together enabled participants to educate researchers on the local issues and context, whilst themselves learning both about AMR and the filmmaking process. In creating this dynamic, the focus of the project was on enabling genuine knowledge exchange [30], where participants felt a sense of power over the messages being carried forwards into video outputs.

Participants continued to share and discuss the videos beyond the project, illustrating a sense of ownership of the materials by participants. The chosen methods of sharing videos were frequently via ‘analogue’ means — i.e. physical showcasing events and accompanying discussions with key stakeholders and wider community members — rather than social media/online platforms. While there was some discussion of the potential of such platforms, in practice the main route the participants chose to share the films was via face-to-face encounters. This would seem to illustrate, perhaps, the participants sense that the films needed to be carefully curated via in-person contextualisation. The showcasing events might not have been prioritised, if this had not been the case, and participants had felt that they could have simply released the films via social media, thereby perhaps reaching more people but without being able to physically engage with their audience. Participants did, however, later decide to use the YouTube platform to share their films, after the showcasing event, to share their films beyond their immediate community. However, this was largely still as a way of enhancing face-to-face encounters (e.g. between HERDi and policymakers) that the community filmmakers themselves were not able to attend.

Echoing the impact upon the community, policy makers were able to see both the issue of AMR as locally impactful and to see community members as capable of creating powerful tools for communication that can also bring new insights to this topic. Again, here, the value of creating what was perceived to be a surprising and impressive output influenced perceptions of the community. Policy makers were able to see community members as experts, capable of generating a clear set of messages through digital communication tools. One example of this type of advocacy in action, where community members themselves could in fact attend, occurred at a 2019 event held in Kathmandu, Nepal. The CE4AMR network (Community Arts for AMR), based at the University of Leeds, held an event specifically to link researchers and policy makers across relevant AMR spaces and approaches. As part of the event, CARAN workshop participants presented their films to national-level Ministry of Health officials and international researchers. Participants were invited to speak at this event, demonstrating a clear link to the new National Action Plan (NAP) which was under development in 2019 and being worked on by many of the attendees of this meeting.

### Limitations

While the PV method has the potential to amplify the voices of marginalised communities and build lines of communication between different community stakeholders, there are also some limitations to reflect on. Achieving empowerment requires sustained interaction between the participants and outside stakeholders [21]. While the CARAN project helped to facilitate these connections, time is needed to evaluate whether these lines of communication remained open and useful to the community. The CARAN project, though insightful, only focussed on a small area within a municipality. Local policymakers did reflect on how the videos might help them to better understand their communities, but it would be difficult to attribute any future policy-level change to the outcomes of this project alone given its size. One further limitation in using PV within a LMIC setting lies in accessibility; both the process of creation and dissemination require access to facilitators, technology and internet. The CARAN films are available via YouTube for viewing, but this would require that potential audiences have access to internet and appropriate devices; both of which might not be accessible in the setting. Moreover, at least one showcasing participant noted (themselves a professional film producer), the videos would need a higher quality production value if they were to gain more mainstream terrestrial distribution and thus a greater audience (*Participant in Chandragiri showcasing FGD*).

## Conclusions

This study exemplifies how video production can function as a tool to share information within communities, and speak directly with policymakers, on the specific topic of AMR. After engaging in a PV project, community members in Nepal reported a sense of pride, confidence and empowerment, allowing them to make what they considered to be a significant contribution to their communities. The videos produced were shared in such a way as to instigate further conversations in order raise awareness, and promote behaviours that can mitigate the impact, of AMR in Nepal. One should not, of course, overstate the potential impact of PV. As reflected on by participants, videos produced by community members are not necessarily tools to be used in isolation. Instead, such outputs might become components of larger advocacy initiatives. Further research should focus on unpacking the potential impact community-made videos can have on community-level and policy-maker perspectives of community issues, as well as the longer-term consequences of participation in such projects and the potential ramifications of creating community champions in addressing AMR.

## Electronic supplementary material

Below is the link to the electronic supplementary material.

Supplementary Material 1: Participant Characteristics in each setting contains tabular information on CARAN project participants from both study sites; Chandragiri and Lokanthali.

## Data Availability

The data used and analysed during this study are available from the corresponding author on reasonable request. Informed consent to publish identifying information or images was obtained from the study participants.
