# Supplementary material for: Using participatory video to generate active agents of change at community level to address the drivers of antimicrobial resistance in two settings in Nepal
Source: BMC Public Health. 2025 Mar 25;25:1137. doi: 10.1186/s12889-024-21181-6 (PMC11938728; doi:10.1186/s12889-024-21181-6)
Supplement: Supplementary file 1 — Supplementary Material 1: Participant Characteristics in each setting contains tabular information on CARAN project participants from both study sites; Chandragiri and Lokanthali. [file 12889_2024_21181_MOESM1_ESM.docx]

| **Lokanthali Participants** | |
| --- | --- |
| **Gender** | |
| Female | 5 |
| Male | 5 |
| **Age Ranges** | |
| 20-30 | 3 |
| 30-40 | 3 |
| 40-50 | 3 |
| 50-60 | 1 |
| **Caste/Ethnicities** | |
| Dangol/Janajati (Newar)  Shrestha/Janajati (Newar)  Aryal/Brahmin  Dangol/Janajati (Newar)  Kutu/ Janajati (Newar)  Dahal/  Brahmin  Neupane/Brahmin  Gurung/Ethnic  Shahi/Chhetri  Hona/  Janajati (Newar) | |
| **Occupations** | |
| Agriculture/business  Pharmacy-Health Post  FCHV (female community health volunteer)  Agriculture/business  Agriculture/business  Animal husbandry-Cow farm  Housewife  Housewife  Pharmacist  Veterinarian [owns veterinary shop] | |
| **Education levels** | |
| Secondary  SLC  Grade 8  Higher Secondary (grade12)  Informal  Grade 9  SLC/Grade 10  B. Pharm | |

**Additional File 1: Participant characteristics in each setting**

| **Chandragiri Participants** | |
| --- | --- |
| **Gender** | |
| Female | 6 |
| Male | 4 |
| **Age Ranges** | |
| 20-30 | 3 |
| 30-40 | 2 |
| 40-50 | 4 |
| 50-60 | 1 |
| **Caste/Ethnicities** | |
| Newar/Janajati  Brahmin  Sahi | |
| **Occupations** | |
| Agro shop  Female community health volunteer (FCHV)  Farmer  Small Poultry farm (chicken)  Animal husbandry (Cow farm)  Housewife  Pharmacist | |
| **Education Levels** | |
| Grade 8  Grade 10  Grade 12  Informal  Grade 9  Pharmacy | |
